# Phase i study of 'dose-dense' pemetrexed plus carboplatin/radiotherapy for locally advanced non-small cell lung carcinoma

**DOI:** 10.1186/1748-717X-6-17

**Published:** 2011-02-16

**Authors:** Xinglei Shen, Albert DeNittis, Maria Werner-Wasik, Rita Axelrod, Paul Gilman, Thomas Meyer, Joseph Treat, Walter J Curran, Mitchell Machtay

**Affiliations:** 1 Department of Radiation Oncology, Kimmel Cancer Center, Jefferson Medical College of Thomas Jefferson University, Philadelphia, USA; 2 Department of Radiation Oncology, Lankenau Hospital and Lankenau Institute for Medical Research, Main Line Health System, Pennsylvania, USA; 3 Department of Medical Oncology, Kimmel Cancer Center, Jefferson Medical College of Thomas Jefferson University, Philadelphia, USA; 4 Department of Hematology/Oncology, Lankenau Hospital and Lankenau Institute for Medical Research, Main Line Health System, Pennsylvania, USA; 5 Eli Lilly, Inc., Indianapolis, USA; 6 Department of Radiation Oncology, Emory University Hospital, Atlanta, USA; 7 Department of Radiation Oncology, University Hospitals, Case Medical Center, Cleveland, USA

## Abstract

**Background:**

This phase I study investigates the feasibility of carboplatin plus dose-dense (q2-week) pemetrexed given concurrently with radiotherapy (XRT) for locally advanced and oligometastatic non-small cell lung cancer (NSCLC).

**Methods:**

Eligible patients had Stage III or IV (oligometastatic) NSCLC. Patients received XRT to 63 Gy in standard fractionation. Patients received concurrent carboplatin (AUC = 6) during weeks 1 and 5 of XRT, and pemetrexed during weeks 1, 3, 5, and 7 of XRT. The starting dose level (level 1) of pemetrexed was 300 mg/m^2^. Following the finding of dose limiting toxicity (DLT) in dose level 1, an amended dose level (level 1A) continued pemetrexed at 300 mg/m^2^, but with involved field radiation instead of extended nodal irradiation. Consolidation consisted of carboplatin (AUC = 6) and pemetrexed (500 mg/m^2^) q3 weeks × 2 -3 cycles.

**Results:**

Eighteen patients were enrolled. Fourteen patients are evaluable for toxicity analysis. Of the initial 6 patients treated on dose level 1, two experienced DLTs (one grade 4 sepsis, one prolonged grade 3 esophagitis). There was one DLT (grade 5 pneumonitis) in the 8 patients treated on dose level 1A. In 16 patients evaluable for response (4 with oligometastatic stage IV disease and 12 with stage III disease), the median follow-up time is 17.8 months. Thirteen of 16 patients had in field local regional response. The actuarial median survival time was 28.6 months in all patients and 34.7 months (estimated) in stage III patients.

**Conclusions:**

Concurrent carboplatin with dose-dense (q2week) pemetrexed at 300 mg/m^2 ^with involved field XRT is feasible and encouraging in patients with locally advanced and oligometastatic NSCLC.

**Trial Registration:**

ClinicalTrials.gov NCT00330044

## Background

Concurrent chemoradiation has been established as the standard of care for non-operable stage III non-small cell lung cancer (NSCLC) [1-4]. With this approach, the median survival time is approximately 17 months and about 15% of patients survive 5+ years. Concurrent combined modality therapy has improved survival over single modality or sequential therapy[1-4], but overall outcomes remain poor.

The optimal chemotherapy regimen to use with concurrent radiation therapy remains uncertain. Initial studies of concurrent treatment have used cisplatin plus a second drug given at near-systemic doses for two cycles during RT [1-4]. No platinum based doublet has clear proven superiority over other regimens. These combinations have significant toxicity with high rates of esophagitis, nausea/vomiting, and myelosuppression.

Alternative less toxic chemotherapy drugs and schedules, most notably weekly carboplatin/paclitaxel regimens have been extensively studied [5-8]. This regimen has been criticized because safe and feasible "radiosensitizing" doses of carboplatin (AUC = 1.5 to 2) and paclitaxel (45-50 mg/m2) are well below the dose intensities considered independently active against NSCLC[9]. Thus, while this regimen may have excellent radiosensitization properties, it likely has little effect on tumor populations outside of the radiation portal. The solution has been to combine concurrent chemoradiotherapy with induction [8,10] or consolidation [4,5] chemotherapy given at systemic doses. This approach necessarily delays the initiation of either local or systemic therapy.

In contrast, the new cytotoxic drug pemetrexed has independent activity against NSCLC and reduced toxicity [11,12], and may be feasible to deliver at near-systemic doses with concurrent radiotherapy[13]. Pemetrexed belongs to the antimetabolite class of antineoplastic drugs. It targets multiple molecules within the folate metabolism pathway, including thymidylate synthase and dihydrofolate reductase. Preclinical data support the hypothesis that pemetrexed serves as a radiosensitizer in addition to having independent activity against NSCLC *in vitro *and *in vivo *[14,15]. A large randomized trial comparing pemetrexed to docetaxel in second line treatment of metastatic NSCLC showed similar response and survival with a more favorable toxicity profile for pemetrexed [16]. In the first line chemotherapy setting for advanced NSCLC, a randomized trial showed that pemetrexed in combination with cisplatin resulted in equivalent survival to gemcitabine with cisplatin [17].

Preliminary results of several studies testing pemetrexed plus radiotherapy have been presented. A phase I trial showed that pemetrexed at a dose of 500 mg/m2 q3weeks (Weeks 1, 4, and 7) could be combined with a full course of standard radiotherapy[13]. Recent update from the CALBG trial #30407 showed that systemic dose pemetrexed may be combined with systemic dose carboplatin (AUC = 6) q3weeks with concurrent radiation to 70 Gy with acceptable toxicities. Efficacy data presented at ASCO 2009 is encouraging, with a median survival time of 22.3 months[18].

The modest toxicity profile of pemetrexed led us to consider whether further intensification of pemetrexed during chemoradiotherapy could be accomplished, with the long-term goal of improving local and distant control. This strategy has successfully improved outcomes in node-positive breast cancer [19,20], aggressive non-Hodgkin's lymphoma[21], and ovarian cancer[22]. We designed and initiated a pilot (phase I) feasibility trial of dose-dense (q2-week) pemetrexed with systemic dose carboplatin and concurrent radiotherapy in the treatment of locally advanced and oligometastatic NSCLC.

## Methods

This was a prospective, investigator-initiated clinical trial, approved by the scientific Clinical Research Committee of the Kimmel Cancer Center at Thomas Jefferson University as well as the Internal Review Board (IRB) of Thomas Jefferson University (TJU). The study was also approved by the IRB of the participating medical center, Lankeanu Hospital (Lower Merion, PA), a member of the Jefferson Health System. Eli Lilly Inc. supported the study with a grant to Thomas Jefferson University; however, the study was written, conducted and analyzed by TJU and Lankenau investigators and sponsored by TJU, independently from any corporate entity. The study was monitored by the Kimmel Cancer Center's Data and Safety Monitoring Board in addition to the investigators.

### Patients/Eligibility

This study was available to patients with locally advanced NSCLC who required definitive full dose radiotherapy as part of their treatment plan. This included stage IIIA, IIIB and oligometastatic stage IV (without diffuse hematogenous metastases) NSCLC. Patients with stage IV NSCLC were only eligible if they had bulky local-regional disease deemed to require high dose local radiotherapy and no symptoms from their extrathoracic disease.

Other eligibility requirements included Zubrod performance status 0-1, absence of severe (>10%) weight loss, FEV1 >1000 cc, serum creatinine < 1.5 mg/dl, serum bilirubin < 1.5 mg/dl, SGOT < 1.5 times institutional upper limits of normal, hemoglobin >8.0 g/dl, ANC >2000 cells/mm^3^, platelets > 100,000 cells/mm^3^, and no recent (< 6 months) myocardial infarction, unstable angina, congestive heart failure or uncontrolled arrhythmia. Exclusion criteria also included prior chemotherapy for lung cancer and/or prior thoracic radiotherapy that would result in field overlap.

### Radiotherapy

Radiotherapy (RT) planning via 3-dimensional, CT-scan based planning was required. Intensity modulated radiation therapy (IMRT) was not used. The choice of field arrangements was left to the discretion of the radiation oncologist, and typically consisted of two to four conformally planned, coplanar fields designed to minimize irradiation of the spinal cord and contralateral lung. Respiratory gating was not used. The protocol-specified dose of radiotherapy to tumor as defined by CT (and PET scan where appropriate) was 63 Gy, given in conventional (1.8-2 Gy) once daily fractionation.

The initial protocol design (Dose Level #1) was to irradiate a large volume to 45 Gy, followed by a cone-down to the gross tumor (plus a small margin) for an 18 Gy boost. This initial volume would include the gross tumor plus a generous margin (at least 2 cm) and the comprehensive bilateral mediastinal nodal space (from the thoracic inlet to at least 5 cm below the carina). In some cases, radiotherapy fields included the inferior mediastinal nodes to the crus of the diaphragm (if subcarinal nodes were involved) and/or contralateral hilar nodes (if bilateral mediastinal nodes were involved), based on the principle of irradiating at least one echelon of lymph nodes beyond that known to be grossly involved.

The protocol was subsequently amended in response to two DLTs to require the use of involved field irradiation from the start of radiotherapy (Dose Level #1A). Involved field radiotherapy included areas positive by CT scan and/or PET scan, with an option to include areas located geographically between two involved areas (e.g. inclusion of the ipsilateral hilum if the adjacent mediastinal nodes are involved). Contralateral mediastinal, contralateral hilar and supraclavicular nodes were no longer electively irradiated.

### Chemotherapy/Dose Escalation Plan

The study was designed to use a fixed dose of carboplatin (AUC = 6, based upon the Cockroft-Gault formula), during Weeks 1 and 5 of radiotherapy (preferably during Day 1 or 2 of those weeks). There were no plans to alter this dose/schedule.

The pemetrexed design of the study was to administer this drug on a biweekly basis (Weeks 1, 3, 5, and 7) during radiotherapy; on Weeks 1 and 5 it would be given together on the same day with carboplatin. The starting dose of pemetrexed for the study was 300 mg/m^2^, with plans to dose escalate to 400 and 500 mg/m^2 ^in subsequent patients based upon analysis of feasibility and toxicity (these dose escalations did not successfully occur).

Dose/schedule modifications were allowed for toxicity. If grade 3-4 neutropenia/thrombocytopenia and/or grade 3 non-hematologic toxicity occurred, all agents (RT, carboplatin, radiotherapy, and pemetrexed) were to be held for 1-2 weeks. When toxicity resolved to Grade 0-1, treatment was to be resumed with a reduction in pemetrexed by 50 mg/m2 (i.e. from 300 mg/m2 to 250 mg/m2). If a second episode of Grade 4 hematologic or grade 3 non-hematologic toxicity were to occur, this would be considered a DLT and the patient removed from study.

Patients who successfully completed carboplatin/pemetrexed and concurrent radiotherapy were allowed to continue on to consolidation carboplatin/pemetrexed after recovering from acute effects of chemoradiotherapy. The consolidation regimen consisted of 2-3 cycles of carboplatin (AUC = 6) and pemetrexed (500 mg/m^2^) q3 weeks. Growth factor (G-CSF or GM-CSF) support was recommended.

Patients were given a subcutaneous injection of B12 (1000 mcg) before starting study treatment and once per month while on study. Folic acid (1000 mcg daily) was also prescribed starting Day 1.

### Study Endpoints/Analysis plan

The primary endpoint of the study was dose-limiting toxicity (DLT), defined as any one of the following serious adverse events (SAE's) as determined by the study investigators and medical monitor to be the result of study treatment:

1. Death within 30 days after the completion of radiotherapy or within 90 days of start of radiotherapy.

2. Grade 4 non-hematologic toxicity occurring during or within 30 days after the completion of radiotherapy or within 90 days of start of radiotherapy.

3. Grade 3 pulmonary toxicity within 90 days after the completion of chemoradiotherapy.

4. Prolonged (>14 days) grade 3 esophagitis 30 days after the completion of radiotherapy or within 90 days of start of radiotherapy preventing the patient from being able to proceed with anti-cancer treatment.

5. Inability to complete at least 54 Gy of thoracic radiotherapy due to toxicity

The Kimmel Cancer Center of Thomas Jefferson University assigned an independent medical monitor to review SAE's with the study investigators and help determine if/when a DLT occurred and if pemetrexed dose may be escalated (or de-escalated) per study protocol.

The statistical plan called for dose escalation from Dose Level #1 (300 mg/m2) to Dose Level #2 (400 mg/m2) if/when none of the first three or one of the first six patients enrolled and evaluable experienced a DLT. A similar plan was made for further escalation beyond Dose Level #2. If at any given dose level, a second DLT occurred, the study was to be closed to further accrual, discussed with the medical monitor and IRB, and modified in order to assure patient safety.

Secondary endpoints included local tumor response rates, and progression-free and overall survival. Survival times were calculated from date of registration on trial.

## Results

### Accrual/Feasibility

This study enrolled its first patient in April 2006; the final patient enrolled in April 2008. There were several time periods where the study was closed for safety/toxicity assessment.

Of the 18 patients accrued, two patients were never treated with study chemoradiotherapy and are not evaluable for any study endpoints. Both patients were found to be fully eligible, signed informed consent, and were enrolled using our institution's registration mechanism. One patient withdrew consent and switched to non-protocol chemoradiotherapy; the second developed a GI bleed and severe anemia prior to receiving any study medication.

Of the remaining 16 patients, two patients are not evaluable for the primary study endpoint (determination of DLT). These two patients (Pts 9 and 10) were enrolled onto Dose Level #2 (400 mg/m2 pemetrexed), based upon what initially appeared to be a favorable toxicity profile among the first six evaluable patients in Dose Level #1. However, as patients #9 and 10 were beginning treatment, a delayed DLT in Dose Level #1 occurred (prolonged esophagitis/weight loss requiring feeding tube). After discussions among the investigators and medical monitor, they were offered the option of withdrawing or continuing with a reduced pemetrexed dose of 250 mg/m2 (Dose Level # -1). These patients opted to continue at the reduced pemetrexed dose. Of note, these two patients completed therapy on schedule with no significant non-hematologic toxicities.

### Patient characteristics

Patient characteristics are shown in Table 1. The median patient age was 70. No patient was considered a candidate for surgical resection. The median FEV1 was 2.00 L. Most patients had stage III disease (6 stage IIIA; 6 stage IIIB) and 4 patients had oligometastatic (3 brain, 1 bone) stage IV disease. Histology was squamous in 3 patients, adenocarcinoma in 9 patients, and not-otherwise-specified (NOS) in 4 patients.

**Table 1:** Patient Characteristics and Treatment Delivery

Patient	Age	Stage	Histology	Dose Level	XRT dose/Tx time (Gy/days)	Concurrent Pemetrexed Dose Received (mg/m^2^)
**1 TJ**	55	IIIB	Ad	1	63/45	1150

**2 ST**	72	IIIB	Sq	1	63/48	900

**3 LM**	64	IIIA	Ad	1	39.6/33*	900

**4 SR**	45	IIIB	Ad	1	59.4/57	1200

**5 MW**	54	IV	Ad	1	63/47	1200

**6 SR**	75	IIIA	Ad	1	63/51	900

**7 PH**	68	IIIB	NOS	2/-1	63/55	1300

**8 JD**	72	IIIB	Ad	2/-1	63/51	1150

**9 JB**	78	IIIB	NOS	1A	63/47	900

**10 BS**	61	IV	NOS	1A	63/49	1200

**11 ES**	63	IV	Ad	1A	63/48	900

**12 JB**	83	IIIA	Sq	1A	63/53	1200

**13 EH**	73	IV	Ad	1A	63/54	1200

**14 ML**	74	IIIA	Ad	1A	63/47	1200

**15 CN**	74	IIIA	Sq	1A	63/49	1200

**16 UG**	62	IIIA	NOS	1A	63/48	1200

*Pt suffered neutropenic fever/sepsis requiring treatment discontinuation.

Histology: Ad = Adenocarcinoma; Sq = Squamous; NOS = Not-otherwise-specified

### Treatment compliance

Fourteen patients were evaluable for the study's primary endpoints of feasibility and assessment of DLT; all these patients received both doses of carboplatin (AUC = 6), and 13 of 14 patients completed their full course of radiotherapy. All 14 patients received at least 3 of four planned courses of pemetrexed (7 of 14 received all four courses; the other 7 missed one course due to neutropenia and/or anemia). Of the 13 patients who completed radiotherapy, 8 received at least two cycles of adjuvant chemotherapy and 5 did not (2 developed progressive disease, 2 had dose limiting toxicities precluding further therapy, and 1 developed a pulmonary embolism).

### Toxicity

Of the six patients enrolled into Dose Level #1, two patients developed DLT. One DLT was neutropenic fever, sepsis and multi-organ failure, which appeared to arise in the setting of colonic perforation at the site of chronic diverticulitis. The patient recovered after urgent surgery and a prolonged hospital course, but was unable to resume any anti-cancer treatment. The second DLT was a delayed and prolonged grade 3 esophagitis after completion of concurrent chemoradiation requiring feeding tube placement, preventing the patient from receiving any additional anti-cancer treatment. Based on these two DLTs, Dose Level #1 was considered infeasible as originally designed and the study was amended utilizing reduced radiotherapy fields.

Dose Level #1A enrolled eight patients evaluable for toxicity. One patient suffered a DLT: an 83 year-old man with significant underlying COPD developed respiratory decompensation one week after completing chemoradiotherapy, and ultimately died. The treating physicians initially determined that this was 'unrelated' to study treatment and primarily related to age and underlying respiratory insufficiency. However, on comprehensive review by the entire study investigation team and independent medical monitors, it was concluded that the patient's death should be considered 'possibly related' to treatment.

The overall toxicity profile of sixteen evaluable patients is shown in Table 2. The most common toxicity was neutropenia, including 7 cases of grade 3-4 neutropenia. There was one Grade 5 toxicity and one patient with Grade 4 non-hematologic toxicity (neutropenic fever/sepsis). Three patients developed grade 3 esophagitis. One patient developed grade 3-4 fatigue. In Dose Level #1, the rate of any Grade 3 or greater non-hematologic toxicity was 33% (2/6); in Dose Level #1A, the rate of Grade 3 or greater non-hematologic toxicity was 25% (2/8).

**Table 2:** Acute Toxicity

Toxicity	Grade 1-2	Grade 3	Grade 4	Grade 5
Dose Level #1* (N = 6)				

Neutropenia	1	2	1 ^††^	---

Thrombocytopenia	1	1	1 ^††^	---

Anemia	3	1	---	---

Esophagitis	3	1	---	---

Pneumonitis	2	1	---	---

Sepsis	--	--	1 ^††^	--

Fatigue	4	--	---	---

Elevated LFT's	4	1 ^††^	---	---

Elevated Creatinine	1	--	---	---

*Worst Toxicity Overall*	*2*	*3*	*1*	*---*

*Worst non-heme Toxicity Overall*	*4*	*1*	*1*	*---*

				

Dose Level #1A* (N = 8)				

Neutropenia	4	3	1	---

Thrombocytopenia	2	1	---	---

Anemia	6	1	---	---

Esophagitis	5	2	---	---

Pneumonitis	3	---	---	1 ^§^

Sepsis	---	---	---	---

Fatigue	5	---	1 ^§^	---

Elevated LFT's	1	---	---	---

Elevated Creatinine	1	---	---	---

*Worst Toxicity Overall*	*3*	*3*	*---*	*1*

*Worst non-heme Toxicity Overall*	*4*	*1*	*---*	*1*

				---

**Overall Study Population (N = 16) **†				

Neutropenia	5	5	2	---

Thrombocytopenia	3	2	1 ^††^	---

Anemia	9	2	---	---

Esophagitis	8	3	---	---

Pneumonitis	5	1	---	1 ^§^

Fatigue	9	0	1	---

Elevated LFT's	5	1	---	---

Elevated Creatinine	2	---	---	---

*Worst Toxicity Overall*	*5*	*6*	*1 *^††^	*1 *^§^

*Worst non-heme Toxicity Overall*	*8*	*2*	*1 *^††^	*1 *^§^

* Dose Level #1 and Dose Level #1A used the same doses (pemetrexed 300 mg/m2 q2-week); however Dose Level #1 used extended field RT and Dose Level #1A used involved field RT.

† Overall study population included all of the patients treated in Dose Level #1 and Dose Level #1A, as well as two patients who were re-assigned from Dose Level #2 (400 mg/m2) to Dose Level -I (250 mg/m2) because of safety purposes.

†† Single patient with sepsis and multi-organ failure in the setting of acute diverticulitis.

§ Elderly (83-year old) patient with COPD, died from respiratory failure 30 days post-RT.

### Response/Efficacy

A total of 15 patients are evaluable for treatment response and patterns of failure (Table 3). In-field local-regional tumor response (partial or complete response) by RECIST criteria was observed in 14, while the other patient demonstrated stable disease without evidence of in-field progression on serial CT and/or PET scans at 40 months. Three patients developed local-regional recurrence as the first site of failure. One progressed just outside of the radiotherapy portal ('marginal miss') and a second patient progressed in an elective nodal region not treated with involved field radiation. These two patients, each with a small focus of intrathoracic progression, were treated with additional radiotherapy. A third patient who progressed within the treatment field at 22 months was treated with salvage brachytherapy. In all, five patients progressed locally at a median time of 10 months.

**Table 3:** Treatment Response and Outcomes Data

Patient	Dose Level	Initial Local response	Distant Metastases	Survival Status
**1 TJ**	1	NR/SD	None	AWD @ 40 mo.

**2 ST**	1	PR	Diffuse Mets	DOD @ 34 mo.

**3 LM**	1	PR	Diffuse Mets	DOD @ 15 mo.

**4 SR**	1	CR	Diffuse Mets	DOD @ 29 mo.

**5 MW**	1	PR	Mets at presentation	DOD @ 6 mo.

**6 SR**	1	CR	None	NED @ 26 mo.

**7 PH**	2/-1	CR	None	AWD @ 26 mo.

**8 JD**	2/-1	PD*	None	NED @ 27 mo.

**9 JB**	1A	CR	None	NED @ 24 mo.

**10 BS**	1A	PR	Mets at presentation	DOD @ 9 mo.

**11 ES**	1A	PR	Mets at presentation	DOD @ 6 mo.

**12 JB**	1A	*NA*	*NA*	DID/DOC @ 3 mo.

**13 EH**	1A	PR	Mets at presentation	DOD @ 6 mo.

**14 ML**	1A	PR	None	NCRM @ 9 mo.

**15 CN**	1A	PR	None	AWD @ 18 mo.

**16 UG**	1A	CR	None	AWD @ 18 mo.

NR/SD: No Response/Stable Disease

PR: Partial Response

CR: Complete Response

PD*: Progressive Disease (one patient had PD just outside of the radiotherapy field edge)

NA: Not Assessable - one patient is not assessable or evaluable due to early (possibly treatment related) death.

AWD: Alive with Disease

DOD: Died of Disease

NED: No Evidence of (Active) Disease

DID/DOC: Died of intercurrent Disease and/or treatment Complications

NCRM: Non-cancer related mortality (one patient died of a MI while NED)

Three patients (20%) without distant metastases at registration developed distant metastases as the first site of failure at a median time of 5 months. Four additional patients (27%) had oligometastatic disease at presentation, and all four succumbed to progressive systemic metastases at a median time of 6 months.

**Figure 1:**
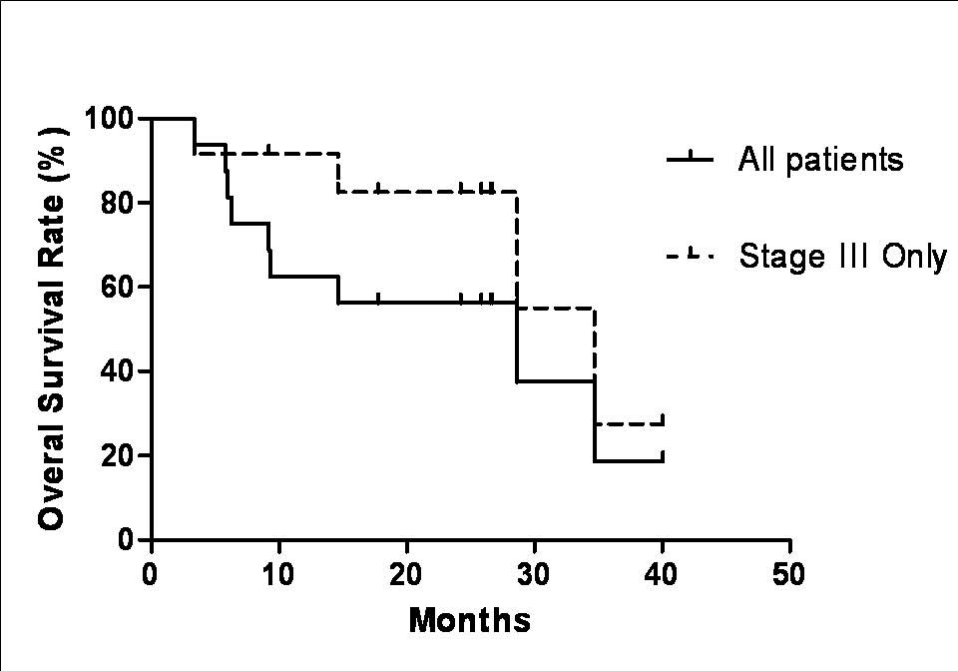
**Kaplan-Meier survival curve**. Median survival time for all patients treated is 28.6 months. For stage III patients, the median survival time was not reached, but estimated at 34.7 months.

There have been nine deaths among the 16 study patients, seven from metastatic NSCLC, one from respiratory failure (due to intercurrent disease and/or treatment complication) and one from an unrelated myocardial infarction. The median follow-up period for all patients is 15.2 months (3 - 40 months) and 24.2 months for surviving patients (12-40 months). The one-year actuarial overall survival rate is 63%, and two-year 56%. Median survival time for all patients was 28.6 months, and among stage III patients it was not reached, but estimated at 34.7 months (Figure 1). Median survival time for oligometastatic patients was 6 months.

## Discussion

We demonstrate the feasibility and safety of combining dose-dense (q2-week) pemetrexed and systemic dose carboplatin (AUC = 6) with radiotherapy for NSCLC. All patients were able to receive both doses of carboplatin and at least three (out of four) doses of pemetrexed with concurrent radiation. All but one patient was able to complete radiotherapy. The main toxicity observed in this study was myelosuppression, primarily manifesting as neutropenia.

Toxicity was decreased by amending the study to mandate the use of smaller (involved-field) radiotherapy treatment plans. We found that 2 of the first 6 patients enrolled in our study suffered non-hematologic DLT's when treated with comprehensive mediastinal irradiation. After changing the radiotherapy planning portion of our study, non-hematologic DLT's occurred in 1 of 8 patients. This patient was an 83 year-old man with severe COPD who died from respiratory decompensation following completion of chemoradiotherapy. We conservatively report this as a DLT although intercurrent disease played a significant role.

When the study was originally designed (in 2005), standard RT consisted of comprehensive mediastinal irradiation to 45-50 Gy followed by a boost to gross tumor to 60-64 Gy, as used in the Radiation Therapy Oncology Group (RTOG) clinical trials [2,23,24]. More recently, data show that outcomes (local control and survival) are not compromised by using involved-field radiotherapy from the start of treatment, and reduced treatment volume may allow the delivery of higher radiotherapy dose [25,26].

In contrast to previous chemoradiation regimens containing reduced radiosensitizing doses of carboplatin and paclitaxel, this study investigated the used of systemic doses of carboplatin (AUC = 6), which has independent activity in NSCLC. The dose dense pemetrexed at 300 mg/m^2 ^also approaches systemic dose of pemetrexed. Although our study was not powered for assessment of anti-tumor efficacy, the results of our survival data are very promising with a median survival time of 28.6 months in all patients, and estimated 34.7 months in stage III patients.

We note that almost all evaluable patients had a clinical local response and at least short term in-field local control of their cancer. Although pathologic assessment of local disease was not performed in our study, our data do support the hypothesis that dose-dense carboplatin/pemetrexed is an effective radiosensitization regimen for definitive therapy of locally advanced non-operative NSCLC.

Despite excellent in field control, progression of disease outside the radiation treatment fields remains exceedingly common. Our study included four patients with oligometastatic diease, and each of these patients quickly progressed systemically with a median survival of only 6 months. Three additional patients developed metastatic disease as the first site of recurrence, and two patients recurred locoregionally outside the treatment field. We hypothesize that this reflects chemoresistance in micrometastatic deposits outside of the radiotherapy fields. With improving local control, the ability to control micrometastatic disease has increasing importance in improving overall survival. It is possible that higher dose intensity of platinum, pemetrexed and/or addition of a third cytotoxic drug could be more effective, although at a cost of higher toxicity.

A recent study by Cullen et al. failed to show an advantage to increased dose intensity of pemetrexed in advanced/metastatic (platinum-refractory) NSCLC[27]. This study compared 500 mg/m2 q3week versus 900 mg/m2 q3week. It is unclear whether dose intensification of pemetrexed using a q2week schedule, or in a less heavily pre-treated population such as ours, might yield different results.

Another strategy might be to add a biologic agent such as a vascular targeting drug or an anti-EGFR agent to our regimen. The Cancer and Leukemia Group B (CALGB) study #30407 investigated in a prospective phase II randomized trial combining carboplatin/radiotherapy and pemetrexed (standard 500 mg/m2 q3-week schedule) with or without cetuximab. Presented in abstract form at the 2009 ASCO national meeting, the carboplatin/pemetrexed/RT arm had a promising median survival time of 22.3 months, but the addition of cetuximab did not result in improved survival with a median survival time of 18.7 months [18].

A complementary strategy may be more careful selection of semi-customized treatments. Randomized studies of single agent pemetrexed in second line chemotherapy treatment of NSCLC and of platinum/pemetrexed in first line treatment of advanced NSCLC demonstrated an improved survival in patients with non-squamous NSCLC, and a worse outcome in patients with squamous histology [16,17]. This difference may be related to increased expression of thymidylate synthase (TS) in squamous cancers or other proteins relevant to the target of pemetrexed[17]. Our study did not collect tissue to perform this analysis, but evaluation of TS will be important to future studies of pemetrexed and radiation in NSCLC.

## Conclusions

Dose-dense (q-2week) pemetrexed at a dose of 300 mg/m2 and carboplatin (AUC = 6) combined with concurrent involved field radiation is feasible. It was not feasible with extended field radiotherapy. Responses are encouraging and this is a suitable platform for further development of future combined modality trials.

## Competing interests

XS, AD, MWW, RA, PG, TM, WJC, MM declare that they have no competing interests.

JT is employed by Eli Lilly, Inc.

## Authors' contributions

MWW, RA, PG, TM, JT, WJC and MM participated in study design. AD, MWW, WJC and MM participated in the radiation therapy of patients. RA, PG, TM participated in the chemotherapy treatment of patients. XS, AD, and MM participated in data collection. XS and MM performed the data analysis analysis.

All authors read and approved the final manuscript.
